# Metastasis Pattern‐Specific Biomarkers and Therapeutic Targets in Gastric Cancer: From Surgical Specimens to Clinical Translation

**DOI:** 10.1002/ags3.70302

**Published:** 2026-10-27

**Authors:** Mitsuro Kanda

**Affiliations:** ^1^ Department of Gastroenterological Surgery Nagoya University Graduate School of Medicine Nagoya Japan

**Keywords:** biomarkers, gastric cancer, metastasis pattern, surgical biospecimens, therapeutic targets

## Abstract

Metastasis has traditionally been investigated and treated as a single manifestation of gastric cancer progression. Although this approach has generated important biological insights and effective systemic therapies, it may obscure route‐dependent vulnerabilities. Lymphatic, peritoneal, and hematogenous metastases require cancer cells to traverse different anatomical routes, overcome distinct barriers, and adapt to different microenvironments. This review therefore distinguishes broadly metastasis‐associated mechanisms from pattern‐associated candidates and reserves pattern‐specific designation for evidence based on direct route comparison, pattern prediction, or route‐selective functional experiments. We examine representative molecular determinants and link their identification in surgical specimens to functional validation, biomarker development, and drug discovery. Evidence is most mature for peritoneal dissemination, whereas lymph‐node‐specific determinants remain less developed. The SYT13/ASO‐4733 program is presented as an illustrative translational case: Intraperitoneal ASO‐4733 has entered first‐in‐human Phase I evaluation, but clinical efficacy has not been established. For any candidate, clinical utility requires independent validation, incremental value beyond established clinicopathological factors, and a defined effect on decision‐making. Rigorously collected, rapidly processed, and clinically annotated surgical biospecimens remain indispensable. Reframing gastric cancer metastasis by route may expand opportunities for precise risk prediction, rational delivery, and biologically coherent early‐phase trials.

## Graphical abstract

Gastric cancer metastasis should be considered according to its anatomical route, because lymphatic, peritoneal, and hematogenous dissemination involve partly distinct molecular mechanisms. This review summarizes metastasis pattern‐associated and pattern‐specific molecules identified using surgical specimens, evaluates their evidence and translational maturity, and discusses their potential as biomarkers and therapeutic targets. Route‐specific investigation may provide new opportunities for clinically meaningful translation from surgical specimens to patient care.

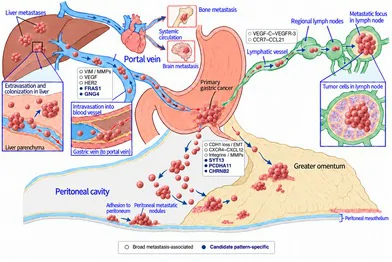

## 1 Introduction

Metastasis is the principal cause of gastric cancer‐related death and commonly presents as lymph node metastasis, peritoneal dissemination, hematogenous metastasis, or a combination of these patterns [1, 2, 3]. For decades, basic research and therapeutic development have largely treated metastatic disease as a single biological and clinical entity. This strategy was reasonable and productive: common features such as epithelial‐mesenchymal plasticity, invasion, immune evasion, and resistance to cell death operate across metastatic sites, and modern systemic therapy has improved outcomes for broadly defined advanced gastric cancer. Nevertheless, different patients preferentially develop different patterns of spread, and each pattern is associated with distinct routes, symptoms, treatment limitations, and natural histories. The broad, site‐agnostic approach may now be reaching an important limit.

A deliberate return to metastatic pattern may create new opportunities. Lymphatic dissemination requires access to lymphatic vessels and adaptation to an immune‐rich nodal niche. Peritoneal dissemination exposes exfoliated cells to anoikis, hypoxia, nutrient deprivation, ascitic fluid, mesothelial surfaces, and the submesothelial matrix. Hematogenous dissemination requires intravasation, survival under circulatory stress, immune escape, arrest in distant capillary beds, extravasation, and colonization of organs such as the liver. Thus, the molecules that determine where a cancer cell can successfully metastasize may not be identical to those that merely indicate aggressive tumor behavior. This distinction is central to developing biomarkers that predict a specific recurrence pattern and therapies that exploit route‐specific vulnerabilities. The distinct anatomical routes are illustrated in Figure 1.

**FIGURE 1:**
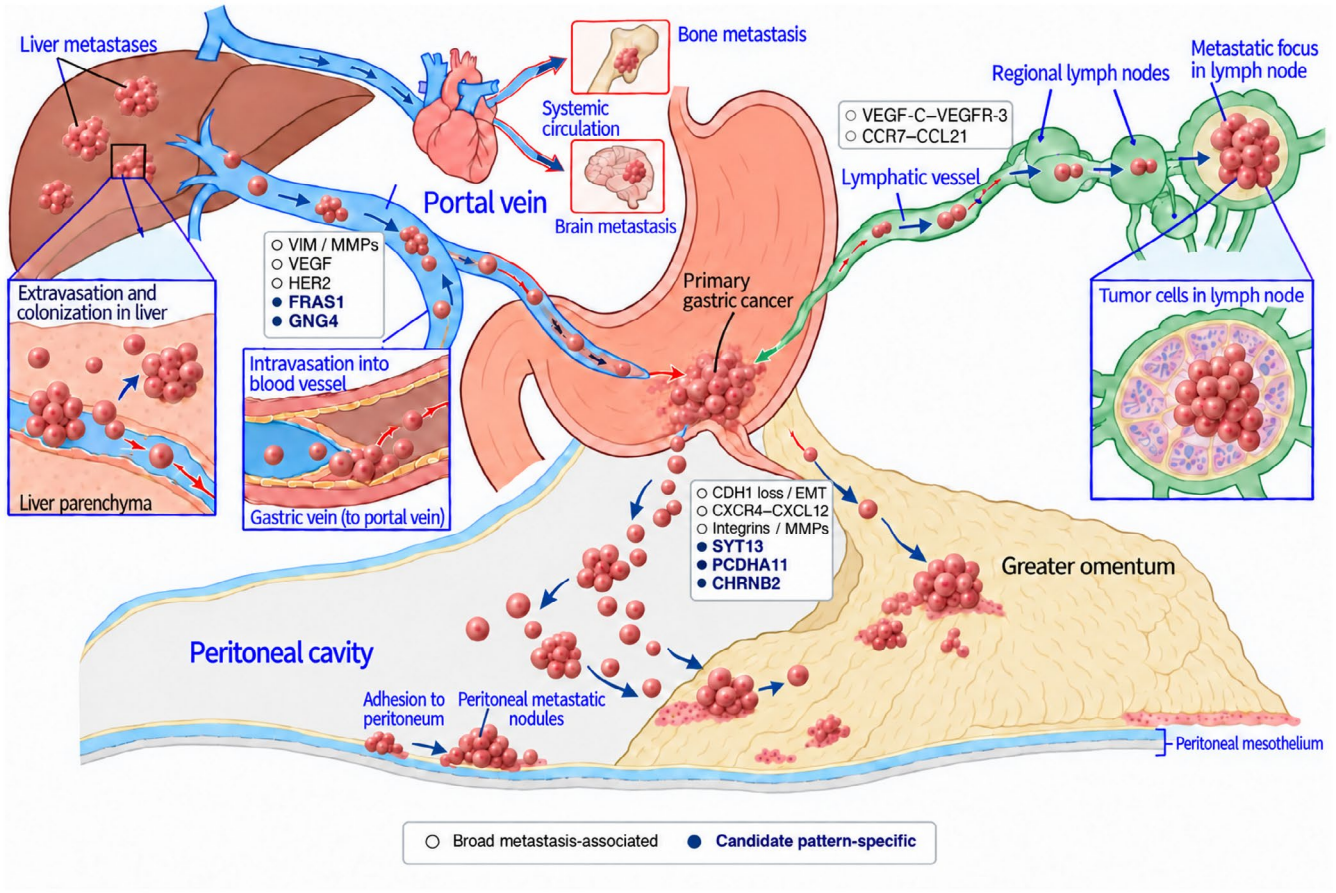
Anatomical routes and evidence categories of gastric cancer metastasis. Candidate molecules are distinguished as broadly metastasis‐associated or supported by route‐selective evidence.

Here, we update our previous reviews of peritoneal and hematogenous metastasis [4, 5] and add lymph node metastasis as a third major pattern. We prioritize candidates supported by direct comparisons among metastatic routes, prediction of a defined recurrence pattern, route‐relevant functional experiments, or translational development. We also place findings from our group within independent international literature and discuss how surgical biospecimens can connect discovery to clinical application.

For this invited narrative review, PubMed was searched through September 2026 using gastric cancer combined with terms for each metastatic route, biomarkers, therapeutic targets, and surgical specimens. Recent English‐language studies were prioritized when they included human biospecimens, direct route comparisons, pattern‐specific recurrence, route‐relevant models, independent validation, or clinical translation. Selection was representative rather than exhaustive.

International literature has largely reviewed peritoneal metastasis, liver metastasis, or precision biomarkers separately [6, 7, 8, 9, 10]. Paired‐genomic analysis further shows that lesions differ by route and may evolve beyond the primary tumor [11]. We compare the three principal routes within one evidentiary framework centered on anatomically informative surgical specimens.

## 2 Defining Metastasis Pattern Specificity

A molecule associated with advanced stage or short survival is not necessarily specific to a metastatic pattern. In this review, pattern‐associated denotes a reproducible relationship with one route that may still reflect general aggressiveness or confounding; pattern‐specific is reserved for evidence from direct comparison with competing routes, prediction of a defined recurrence pattern, or perturbation that selectively alters dissemination in a route‐appropriate model. Analyses should account for histological type, depth of invasion, treatment exposure, and competing risks, and should distinguish malignant‐cell expression from stromal or immune‐cell signals. Independent clinical validation should then be integrated with mechanistic experiments.

Evidence is organized as a translational hierarchy: association, pattern prediction, functional causality, in vivo therapeutic validation, and clinical testing. Actionability additionally requires target engagement, antimetastatic activity, feasible delivery, and an acceptable therapeutic window. Few candidates complete this path. Table 1 therefore separates the observed clinical evidence from the proposed application and states the highest stage reached, preventing exploratory correlations from being presented as established drivers or clinically actionable targets.

**TABLE 1:** Representative metastasis pattern‐associated and candidate pattern‐specific molecules in gastric cancer.

Metastatic pattern	Molecule or axis	Physiological function	Oncological function	Evidence in clinical biospecimens	Potential application	Stage of translation	References
Lymph node metastasis	VEGF‐C/VEGF‐D–VEGFR‐3	Lymphatic development; fluid homeostasis; immune‐cell trafficking	Supports lymphangiogenesis, lymphatic entry, migration, invasion, and survival	Primary tumor (mRNA/IHC) Regional lymph nodes and lymphatic‐vessel density Serum/plasma association studies	Tissue biomarker Liquid biomarker Investigational antibody/other therapy	Clinical association; functional validation	[12, 13]
	CCR7–CCL21–CLDN11	Immune‐cell homing to lymphoid tissue; specialized tight‐junction barriers	Promotes proliferation, chemotaxis, invasion, and lymph‐node homing	Paired primary tumors and lymph‐node metastases (RNA‐seq) Primary and metastatic lesions (IHC)	Tissue biomarker Investigational antibody/other therapy	Clinical association; functional validation	[14, 15]
	Exosomal lncAKR1C2 / pep‐AKR1C2	Steroid/prostaglandin metabolism; microprotein function remains incompletely defined	Exosomal export promotes metastatic competence and lymphatic‐endothelial reprogramming	Primary tumor Plasma‐derived exosomes	Tissue/liquid biomarker Investigational nucleic‐acid/other therapy	Clinical association; functional validation	[16]
	circPAK2–IGF2BP–VEGFA	Cytoskeletal regulation, cell survival, vascular homeostasis, and RNA regulation	Stabilizes VEGFA‐related transcripts, enhancing proliferation, migration, invasion, and nodal metastasis	Primary tumor Resected nodal metastases Extracellular‐vesicle RNA, where evaluated	Tissue/liquid biomarker Investigational nucleic‐acid/other therapy	Clinical association; functional validation	[17]
Peritoneal metastasis	CDH1 loss and EMT programs	Epithelial adhesion and tissue architecture; developmental and repair plasticity	Increases detachment, motility, invasion, and anoikis resistance	Primary tumor (histology/IHC) Germline/tumor DNA Peritoneal metastatic tissue	Tissue biomarker Exploratory therapeutic pathway	Pattern‐associated; not route‐specific	[4, 6, 18]
	CXCR4–CXCL12	Stem‐cell retention, leukocyte trafficking, organ development, and repair	Promotes survival, chemotaxis, invasion, and peritoneal homing	Primary tumor Ascites/peritoneal fluid Peritoneal nodules	Tissue/liquid biomarker Investigational antibody/other therapy	Clinical association; functional validation; route specificity unproven	[4, 19]
	Integrins, CD44, MMPs, and TGF‐β	Cell–matrix adhesion, matrix turnover, tissue homeostasis, and wound repair	Promotes mesothelial/ECM adhesion, matrix degradation, invasion, and plasticity	Primary tumor Ascites/peritoneal lavage Peritoneal metastatic tissue	Tissue/liquid biomarker Exploratory therapeutic pathway	Pattern‐associated; broad metastatic mechanisms	[4, 6]
	PCDHA11	Protocadherin‐family cell recognition; organ‐level role incompletely characterized	Promotes proliferation, invasion, spheroids, and stem‐like properties through AKT/mTOR, Wnt, and JAK/STAT	Primary tumor and expression datasets Surgically derived models	Tissue biomarker Investigational nucleic‐acid therapy	Clinical association; functional and in vivo therapeutic validation; single‐group evidence	[20]
	CHRNB2	Nicotinic acetylcholine‐receptor signaling	Promotes proliferation, survival, invasion, adhesion, stem‐like properties, and drug resistance	Primary tumor (transcriptome/IHC) Metastatic‐potential‐stratified surgical specimens	Tissue biomarker Investigational antibody therapy	Clinical association; functional and in vivo therapeutic validation; single‐group evidence	[21]
	SYT13	Atypical synaptotagmin implicated in membrane/vesicle trafficking	Promotes adhesion, survival, migration, invasion, and peritoneal metastatic capacity	Primary tumor (mRNA/IHC) Peritoneal lavage (SYT13 mRNA ± CEA) Peritoneal metastatic tissue	Tissue/liquid biomarker Investigational nucleic‐acid therapy	Clinical association and prediction; functional/in vivo validation; ongoing Phase I testing—efficacy unestablished	[22, 23, 24, 25, 26]
	Exosomal galectin‐3	Glycan recognition, adhesion, immune regulation, inflammation, and fibrosis	Exosomal release supports implantation and remodels mesothelial/stromal cells	Tumor/peritoneal lesions Ascites‐derived EVs Plasma EVs, where evaluated	Tissue/liquid biomarker Investigational antibody/other therapy	Clinical association; functional validation	[27]
Hematogenous metastasis	VEGF, TYMP, and GLUT1	Angiogenesis, thymidine catabolism, and glucose transport	Supports hypoxic adaptation, glycolytic survival, invasion, and proangiogenic signaling	Primary tumor (mRNA/IHC) Serum/plasma VEGF Selected hepatic metastases	Tissue/liquid biomarker Investigational antiangiogenic/other therapy	Clinical association; broad metastatic mechanisms	[5]
	HER2 (ERBB2)	Epithelial growth, differentiation, and tissue development	Amplification/overexpression drives proliferation, survival, invasion, and resistance	Primary tumor or biopsy (IHC/ISH) Metastatic tissue	Established tissue biomarker Approved systemic therapeutic target	Clinically actionable; association with liver metastasis reported, but route causality unproven	[28, 29]
	FRAS1	Basement‐membrane and extracellular‐matrix organization	Supports proliferation, hypoxic survival, stemness, invasion, and liver metastatic capacity	300‐patient surgical cohort Pattern‐specific transcriptome specimens Liver metastases in discovery analyses	Tissue biomarker Investigational nucleic‐acid/other therapy	Pattern prediction; functional and route‐selective in vivo validation; single‐group evidence	[30]
	GNG4	Heterotrimeric G‐protein signaling	Promotes proliferation, cell‐cycle progression, adhesion, tumorigenicity, and liver‐niche growth	Paired primary/adjacent/liver‐metastatic tissue 300‐patient surgical cohort	Tissue biomarker Investigational nucleic‐acid/other therapy	Pattern prediction; functional and route‐selective in vivo validation; single‐group evidence	[31]
	MFSD4	Putative membrane transport and trafficking	Reduced expression is associated with hepatic metastatic potential; proposed tumor‐suppressive role	Pattern‐selected primary tumors Surgical validation cohort	Tissue biomarker	Clinical association and pattern prediction; limited external validation	[5]
	GPR155	G‐protein‐coupled receptor signaling	Reduced expression is associated with hematogenous recurrence and altered ERK/STAT signaling	Pattern‐selected primary tumors Stage II/III surgical cohort	Tissue biomarker	Clinical association and pattern prediction; functional validation; limited external validation	[5]
	NCPAP3	RNA stability and translational regulation	Expression abnormality has been reported as a hepatic‐metastasis predictor; function remains insufficiently defined	Primary tumor discovery and validation specimens	Tissue biomarker	Clinical association; exploratory prediction; limited functional evidence	[5]
	PAK1–ATF2–miR‐132	Cytoskeletal dynamics and post‐transcriptional regulation	PAK1 activation and reduced miR‐132 promote adhesion, migration, invasion, and hematogenous metastasis	Primary gastric‐cancer tissue Advanced‐stage surgical specimens	Tissue biomarker Investigational nucleic‐acid/other therapy	Clinical association; functional and route‐selective in vivo validation	[5]
	Exosomal integrins and EV cargo including ILK1/CD14	Adhesion, cytoskeletal signaling, innate immune sensing, and EV communication	EV packaging supports organotropic communication and metastatic fitness	Plasma EVs from advanced gastric cancer Primary/metastatic tissue in selected studies	Liquid biomarker Investigational antibody/other therapy	Clinical association; organotropism remains investigational	[32, 33]

*Note:* Potential applications are proposed or investigational unless explicitly described as established. Clinical testing denotes entry into human evaluation and does not imply therapeutic efficacy. “Single‐group evidence” indicates that independent external replication remains limited.

## 3 Molecular Determinants of Lymph Node Metastasis

Lymph node metastasis begins before a cancer cell enters a lymphatic vessel. Tumor‐derived signals expand and remodel peritumoral lymphatics, increase lymphatic endothelial permeability, and may condition the draining node. Cancer cells then invade lymphatic capillaries, migrate with lymph flow, and survive within a node containing stromal, endothelial, and immune‐cell compartments [12, 13]. Lymph nodes can function as both metastatic destinations and potential gateways to systemic dissemination. This cascade differs fundamentally from direct exfoliation into the peritoneal cavity or entry into blood vessels [13].

The VEGF‐C/VEGF‐D‐VEGFR‐3 pathway provides the best‐established framework for tumor lymphangiogenesis [12, 13]. VEGF‐C from cancer or stromal cells promotes lymphatic endothelial proliferation and access to lymphatic channels. The CCR7‐CCL21 axis can add directional migration, and transcriptomic comparison of primary gastric tumors and lymph node metastases proposed a CCL21‐CCR7‐CLDN11 program, although cellular origin and independent paired‐cohort validation remain unresolved [14, 15]. CXCR4‐CXCL12, adhesion molecules, MMPs, and PI3K‐AKT signaling can facilitate nodal spread but also operate in peritoneal and hematogenous dissemination; they are therefore broad metastasis‐associated mechanisms unless route‐selective evidence is shown [4, 5, 10, 19].

Noncoding RNA and extracellular‐vesicle studies are expanding this framework. A microprotein encoded by exosomal lncAKR1C2 was reported to promote lymph node metastasis through metabolic reprogramming, whereas m6A‐modified circPAK2 enhanced VEGFA signaling through IGF2BP proteins [16, 17]. These studies suggest that lymphatic spread depends not only on cell motility but also on metabolic fitness and communication with lymphatic endothelium. Their translational value will depend on validation in prospectively collected plasma and paired nodal tissues.

Evidence for lymph‐node‐specific determinants is less mature than that for peritoneal dissemination. Most studies establish association or lymphangiogenic plausibility rather than prediction against competing routes or route‐selective causality. A clinically useful marker must add information beyond imaging, depth, histological type, lymphovascular invasion, and established pathological staging—for example, by predicting occult nodal disease before endoscopic or limited surgery or by refining perioperative treatment [3, 34]. Therapeutic inhibition must also preserve normal lymphatic function and immune‐cell trafficking. Spatial analysis of the invasive front and lymphatic entry sites may be more informative than bulk expression alone.

## 4 Molecular Determinants of Peritoneal Dissemination

Peritoneal dissemination is a sequential but overlapping process: invasion through the gastric wall, release into the peritoneal cavity, survival in suspension, mesothelial adhesion, submesothelial invasion, and growth with angiogenesis [4, 6, 7, 18]. International reviews have integrated genomic biology with peritoneal‐directed treatment and have emphasized intrinsic tumor programs, tumor‐mesothelial adhesion, and the peritoneal microenvironment [6, 7]. The present review adds an explicit cross‐route evidentiary hierarchy and a surgical‐specimen‐to‐translation framework. Serosal exposure and large infiltrative tumors remain clinical risk features [35]. Loss of epithelial adhesion, EMT‐related plasticity, and matrix degradation facilitate escape, but these broadly metastatic processes should not be labeled peritoneal‐specific without comparative evidence.

Free cancer cells must survive a hostile cavity characterized by loss of anchorage, fluctuating oxygen and nutrients, mechanical movement, and immune surveillance. HIF‐1alpha signaling, PI3K‐AKT pathway activation, CXCR4‐CXCL12 signaling, stem‐like states, and spheroid formation contribute to survival and migration [4, 6]. Malignant ascites is not a passive fluid. It contains tumor cells, cancer‐associated fibroblasts, macrophages, neutrophils, lymphocytes, soluble factors, and extracellular vesicles. Single‐cell and spatial analyses of matched primary tumors, peritoneal lesions, and ascites have identified MUC1‐positive malignant cells, proangiogenic C1Q‐positive macrophages, fibroblast interactions, and treatment‐associated remodeling of the peritoneal ecosystem [36]. Exosomal galectin‐3 and other vesicle‐associated cargo may further prepare the peritoneal microenvironment [27].

Attachment to and invasion through the mesothelial barrier involve integrins, CD44, selectins, matrix metalloproteinases, and reciprocal signaling with mesothelial cells [4, 6]. TGF‐beta can promote mesothelial‐to‐mesenchymal transition and expose a permissive extracellular matrix. Cancer‐associated fibroblasts, omental adipocytes, macrophages, neutrophil extracellular traps, and milky spots contribute to implantation and growth. These observations shift the therapeutic concept from targeting cancer cells alone to disrupting the cancer cell‐mesothelium‐stroma ecosystem [4, 6].

Two additional candidates illustrate complementary drug‐development routes. Protocadherin alpha 11 (PCDHA11), identified as an oncogenic adhesion‐related molecule, supported proliferation, migration, invasion, spheroid formation, and stem‐like properties through AKT/mTOR, Wnt/β‐catenin, and JAK/STAT signaling. Amido‐bridged nucleic acid‐modified antisense oligonucleotides against PCDHA11 suppressed tumor growth in gastric and pancreatic cancer peritoneal metastasis models, as well as in systemic‐metastasis and established‐subcutaneous‐tumor models [20]. The lead sequences nevertheless produced reversible, dose‐ and sequence‐dependent liver injury, underscoring the need to optimize therapeutic index before clinical translation. CHRNB2, a cell‐surface nicotinic acetylcholine‐receptor subunit identified through transcriptomic screening of metastasis‐prone gastric cancers, promoted proliferation, survival, migration, invasion, extracellular‐matrix adhesion, stem‐like properties, and drug resistance through PI3K–AKT and JAK–STAT signaling. A CHRNB2‐targeting monoclonal antibody inhibited tumor growth and prevented omental and mesenteric nodules in a peritoneal metastasis model [21]. Together, PCDHA11 and CHRNB2 show how surgical‐specimen discovery can be matched to nucleic‐acid and antibody modalities, respectively.

SYT13 illustrates how surgical specimens can identify a clinically relevant, pattern‐associated molecule. SYT13 expression in primary gastric cancer was associated with peritoneal recurrence, and SYT13 mRNA in peritoneal lavage fluid provided molecular information beyond conventional cytology [22, 23]. Functional studies support roles in adhesion, invasion, and peritoneal metastatic capacity. Nevertheless, standardized sampling, prespecified thresholds, comparison with serosal invasion, histological phenotype and cytological status, and independent prospective validation are required before clinical utility can be claimed.

SYT13 has progressed from biomarker research to therapeutic development. Amido‐bridged nucleic acid‐modified antisense oligonucleotides targeting SYT13 suppressed peritoneal disease in preclinical models, and toxicological evaluation supported intraperitoneal development [24, 25]. The investigator‐initiated first‐in‐human Phase I study of intraperitoneal ASO‐4733 with S‐1 plus oxaliplatin for gastric or gastroesophageal junction cancer with peritoneal dissemination is ongoing (jRCT2041250021) [26]. Its primary purpose is to evaluate dose‐limiting toxicity, safety, and pharmacokinetics; antitumor activity is exploratory, and efficacy has not been established. This program is presented as a proof‐of‐concept for translation from surgical discovery to route‐directed clinical testing, not as evidence of therapeutic benefit.

## 5 Molecular Determinants of Hematogenous Metastasis

Hematogenous metastasis requires invasion of blood vessels, survival in the circulation, arrest at a distant vascular bed, extravasation, and colonization [5, 37]. EMT‐related proteins, vimentin, matrix metalloproteinases, and growth‐factor signaling contribute to intravasation. In the bloodstream, tumor cells encounter shear stress, immune attack, and anoikis. Platelet coating and multicellular circulating tumor‐cell clusters can provide protection and increase metastatic efficiency [38, 39]. These shared mechanisms establish systemic competence but do not fully explain organotropism.

The liver is a major hematogenous destination for gastric cancer, but portal venous anatomy alone does not determine which tumors colonize it. International synthesis has highlighted vascular invasion, circulating‐cell survival, premetastatic‐niche formation, hepatic microenvironmental adaptation, and potentially targetable signaling as interdependent steps [8, 32, 37, 40]. Tumor‐derived cytokines and extracellular vesicles can modify sinusoidal endothelial cells, Kupffer cells, hepatic stellate cells, and recruited myeloid cells before overt metastasis. This multistep compatibility between seed and soil provides a stronger framework for organotropism than vascular delivery alone [41].

Candidate biomarkers for hematogenous or hepatic metastasis include VEGF, thymidine phosphorylase, GLUT1, HER2, NPM1, and NCPAP3 [5]. HER2‐positive gastric cancer shows a greater frequency of liver metastasis and a lower frequency of peritoneal dissemination in some clinical series, but this may reflect intestinal histology, chromosomal instability, or treatment selection rather than route causality [28, 29]. Plasma extracellular‐vesicle ILK1 and CD14 are associated with organ‐specific patterns but remain investigational [33]. HER2 assessment by plasma ctDNA is likewise emerging rather than established contemporary practice. Genomic comparison of paired metastases shows route‐related evolutionary differences, including greater chromosomal instability in hematogenous lesions, but molecular subtype should not be conflated with a causal determinant of destination [9, 11].

Recurrence pattern‐specific transcriptomics has also yielded experimentally supported drivers of hepatic metastasis. FRAS1 was identified from comparisons centered on liver metastatic recurrence and was evaluated in a 300‐patient surgical cohort. High FRAS1 expression in primary gastric tumors was associated with an increased cumulative incidence of liver recurrence. FRAS1 knockout impaired proliferation, stemness, migration, invasion, resistance to oxygen stress, tumor formation, and metastatic capacity, with involvement of EGFR and PI3K signaling [30]. GNG4 was identified by comparing primary tumor, adjacent gastric tissue, and liver metastasis from patients whose metastasis was confined to the liver, followed by validation in primary tumors from 300 patients. High GNG4 expression was associated with poor survival and liver recurrence; GNG4 loss produced only moderate inhibition in a subcutaneous model but striking suppression in a liver metastasis model, suggesting dependence on the hepatic context [31]. FRAS1 and GNG4 therefore provide unusually direct links among surgical specimens, pattern‐specific prediction, functional causality, and therapeutic‐target nomination.

Therapeutic opportunities include inhibition of angiogenesis, tumor‐platelet interactions, circulating‐cell clustering, extracellular‐vesicle uptake, and premetastatic‐niche formation. HER2‐targeted therapy illustrates actionability of a molecular phenotype that may correlate with a route, not a site‐specific treatment [10, 28, 29, 42]. Future trials should prospectively record metastatic pattern and integrate tissue and liquid biomarkers. Enrichment by route is justified only when analytical validity, independent prediction beyond established factors, and a plausible treatment consequence have been demonstrated.

## 6 Surgical Biospecimens as the Foundation of Metastasis Research

The technologies used to interrogate cancer have changed dramatically, but high‐quality clinical specimens remain the foundation of meaningful metastasis research. Gastric cancer surgery provides access not only to primary tumor and adjacent mucosa but also to the invasive front, regional lymph nodes, peritoneal nodules, liver lesions, peritoneal lavage, ascites, peripheral blood, and occasionally portal venous blood [43]. Paired primary‐metastatic samples and longitudinal specimens before and after therapy are especially valuable. Crucially, every specimen must be linked to detailed pathological findings, treatment exposure, timing, and the actual pattern of recurrence. Without reliable clinical annotation, metastatic specificity cannot be established.

Preanalytical quality is biological quality. Warm ischemia before resection, cold ischemia between resection and stabilization, sampling from necrotic or fibrotic areas, fixation conditions, and repeated freeze–thaw cycles can alter RNA, phosphorylation states, proteins, metabolites, and cellular viability [44, 45]. Peritoneal lavage and blood require standardized volumes, timing, anticoagulants, centrifugation, separation of cellular and fluid fractions, and storage temperatures. The intended analysis should determine whether tissue is snap‐frozen, placed in preservation reagent, fixed for histology, or processed immediately for viable cells and organoids [44, 45]. Time stamps and deviations from standard operating procedures should accompany the sample rather than being reconstructed later [44, 45].

Modern single‐cell sequencing, spatial transcriptomics, multiplex imaging, organoids, patient‐derived xenografts, and liquid biopsy do not replace careful surgical sampling; they increase its value [36, 43, 46, 47, 48]. Sampling the tumor center alone may miss lymphatic invasion or invasive‐front interactions. Bulk analysis of a metastatic lymph node may measure immune cells rather than cancer cells. Ascites collected after treatment may represent an evolved ecosystem rather than the untreated disease [27, 34, 36]. Surgeons are therefore not merely specimen providers. They can define the metastatic phenotype, identify the anatomically informative site, minimize the interval to processing, and integrate molecular observations with the clinical course. This role is essential for translating a statistically associated molecule into a biologically credible target. The translational workflow from surgical specimens to clinical application is summarized in Figure 2.

**FIGURE 2:**
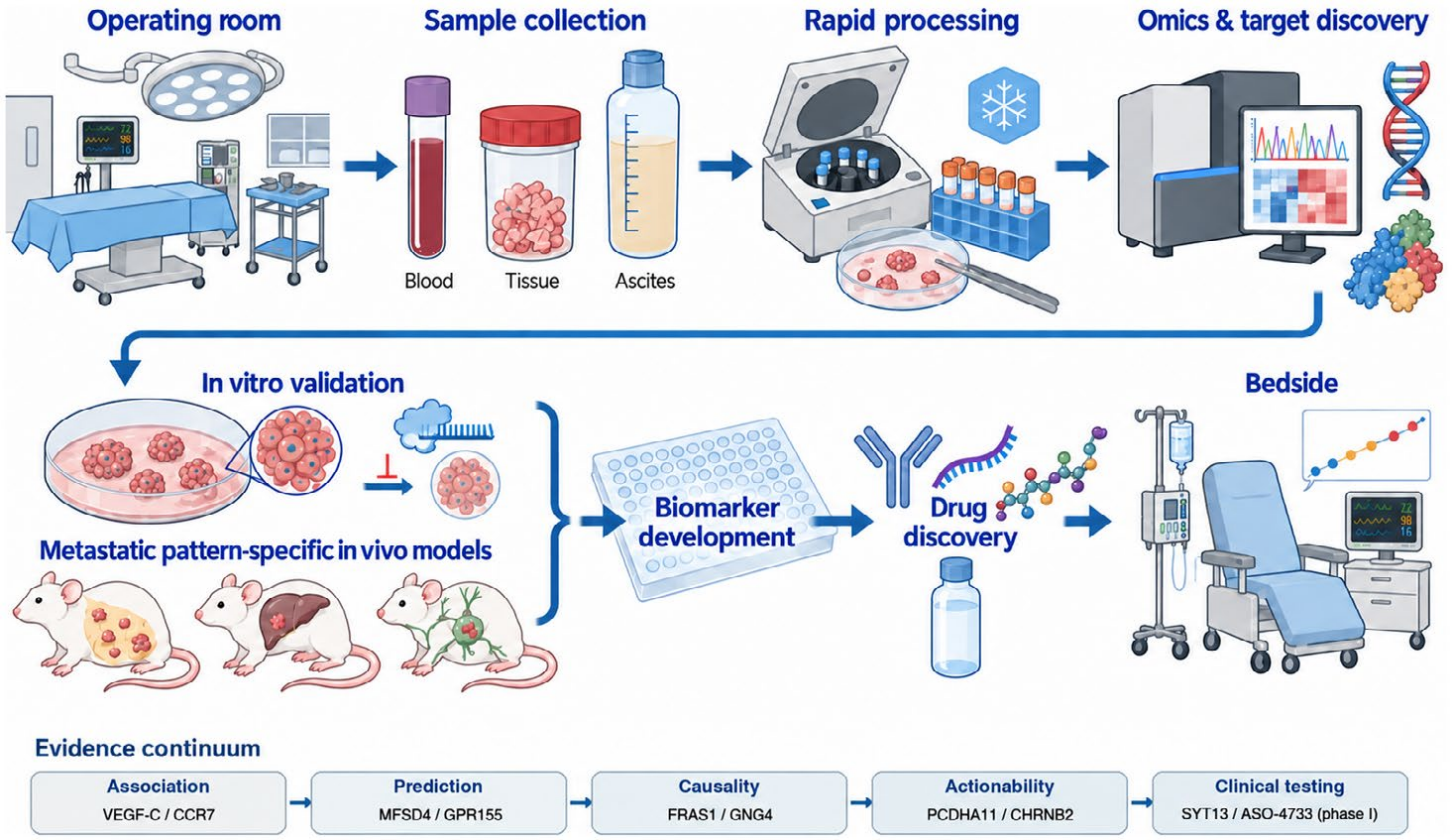
Evidence continuum from surgical specimens to clinical translation. Candidates advance through association, prediction, causality, therapeutic validation, and clinical testing; attrition and reassessment occur at each step.

## 7 From Biomarkers to Therapeutic Opportunities

A metastasis pattern‐specific biomarker can support risk prediction, occult‐disease detection, treatment selection, pharmacodynamic assessment, or preventive‐trial enrollment [46, 49]. Clinical utility, however, requires more than association: a model should be independently validated, calibrated, compared with established factors, and shown to change a decision or improve an outcome. A marker of nodal disease should add to imaging and pathological variables; a marker of peritoneal recurrence should add to serosal invasion, histological phenotype, and cytology. Primary‐tumor immunohistochemistry is spatially informative, lavage samples the at‐risk compartment, and circulating analytes permit serial assessment but may be anatomically nonspecific [23, 33, 46, 47, 49].

Therapeutic modality and delivery route should reflect biology. Antibodies, peptides, siRNA, and antisense oligonucleotides may enable target‐specific intervention, whereas intraperitoneal delivery can increase local exposure for peritoneal disease [20, 21, 24, 25, 26]. A candidate should be called a drug target cautiously until target engagement, route‐relevant in vivo efficacy, delivery feasibility, and safety are demonstrated. Contemporary functional precision medicine increasingly combines molecular profiling with patient‐derived models, but prospective evidence that this strategy improves outcomes in gastric cancer remains limited [10]. Biomarker and drug development should proceed together so that target‐positive patients and pharmacodynamic effects can be identified.

## 8 Limitations and Evidence Gaps

Most candidates remain preclinical or derive from retrospective, single‐center cohorts. Apparent specificity may be confounded by histology, stage, therapy, or sampling; heterogeneity and clonal evolution weaken single‐biopsy inference. Competing routes are often not analyzed, negative replication is rarely reported, and models may bypass the route represented. Many markers fail to improve established prediction, while therapeutic perturbation may lack deliverability or a safe window. These limitations explain why association seldom becomes clinical utility.

## 9 Conclusions

The next phase of metastasis research should combine paired primary and metastatic lesions, serial liquid biopsies, single‐cell and spatial multi‐omics, and phylogenetic reconstruction. Evidence maturity is unequal: peritoneal dissemination includes candidates extending from discovery to clinical testing, hematogenous studies increasingly incorporate organ‐relevant models and paired genomics, and lymph‐node‐specific prediction and intervention remain comparatively underdeveloped. Tumors may also possess several metastatic programs, and therapy may suppress one while selecting another. Prospective route‐annotated cohorts and embedded translational studies are needed to test this dynamic view.

Historically, basic studies and clinical trials have grouped metastatic patterns together. This strategy established common principles and important systemic treatments, but progress may now be constrained by biological heterogeneity hidden within the term metastatic gastric cancer. Separating lymphatic, peritoneal, and hematogenous dissemination can expose distinct dependencies, identify the most informative specimen, and support coherent trial design. The SYT13/ASO‐4733 program illustrates the translational path and its current boundary: first‐in‐human testing has begun, but efficacy remains unknown. Reconnecting meticulous surgical observation with critical, independently validated experimentation may broaden the chance of the next breakthrough.

## Author Contributions

**Mitsuro Kanda:** conceptualization, funding acquisition, writing – review and editing, writing – original draft, visualization.

## Funding

The ASO‐4733 Phase I study (jRCT2041250021) is supported by the Japan Agency for Medical Research and Development (AMED: 24ym0126155h0001).

## Ethics Statement

The author has nothing to report.

## Consent

The author has nothing to report.

## Conflicts of Interest

The author declares no conflicts of interest.

## Data Availability

Data sharing not applicable to this article as no datasets were generated or analysed during the current study.
